# Cluster of Serogroup W135 Meningococci, Southeastern Florida, 2008–2009

**DOI:** 10.3201/eid1601.091026

**Published:** 2010-01

**Authors:** Timothy J. Doyle, Alvaro Mejia-Echeverry, Paul Fiorella, Fermin Leguen, John Livengood, Robyn Kay, Richard Hopkins

**Affiliations:** Department of Health, Miami, Florida, USA (T.J. Doyle); Centers for Disease Control and Prevention, Miami (T.J. Doyle); Miami-Dade County Health Department, Miami (A. Mejia-Echeverry, F. Leguen); Department of Health, Jacksonville, Florida, USA (P. Fiorella, R. Kay); Broward County Health Department, Ft. Lauderdale, Florida, USA (J. Livengood); Department of Health, Tallahassee, Florida, USA (R. Hopkins)

**Keywords:** Meningococcal disease, serogroup W135, bacteria, Florida, outbreak, dispatch

## Abstract

Recently, 14 persons in southeastern Florida were identified with *Neisseria meningitidis* serogroup W135 invasive infections. All isolates tested had matching or near-matching pulsed-field gel electrophoresis patterns and belonged to the multilocus sequence type 11 clonal complex. The epidemiologic investigation suggested recent endemic transmission of this clonal complex in southeastern Florida.

*Neisseria meningitidis* serogroup W135, which generally accounts for <5% of invasive meningococcal disease in the United States, has frequently been associated with foreign travel and is less often associated with outbreaks than other serogroups (*1*). In Florida during 2004–2007, a total of 337 patients statewide were reported to have meningococcal disease; 6 (1.8%) of these were caused by serogroup W135.

## The Study

Meningococcal disease is a reportable condition in Florida. Isolates of *N. meningitidis* from persons with invasive disease are forwarded to the state health department Bureau of Laboratories for serogrouping by slide agglutination. Isolates from outbreaks or clusters are further characterized by pulsed-field gel electrophoresis (PFGE) by using *Nhe*I and *Spe*I restriction enzymes and methods consistent with standard protocols (*2*).

**Figure 1:**
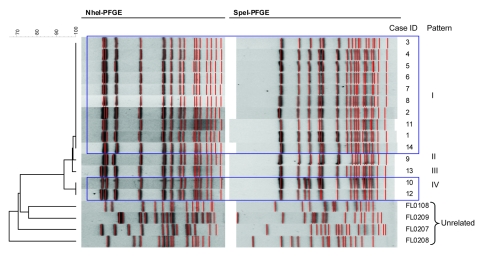
Pulsed-field gel electrophoresis (PFGE) patterns for 14 related and 4 unrelated isolates of *Neisseria meningitidis* serogroup W135, Florida, USA.

From December 2008 through April 2009, we observed an increase of invasive meningococcal disease caused by serogroup W135 in southeastern Florida totaling 13 patients, of whom 9 had indistinguishable PFGE patterns (Figure 1). Isolates from the 4 other case-patients had PFGE patterns differing by 1 (pattern II), 2 (pattern III), and 5 bands (pattern IV), making them >94% related to the dominant pattern. A retrospective review of all 5 W135 isolates in Florida from January 2007 through November 2008 identified 1 additional isolate matching the dominant pattern from a resident of southeastern Florida with illness onset in May 2008. The 4 other isolates from 2007–2008 had PFGE patterns <80% related (Figure 1) and occurred before May 2008 in residents of central and northern Florida.

Of the 14 case-patients with matching or near-matching PFGE patterns (Table 1), 13 denied recent foreign travel. One patient with disease onset in April 2009 was a tourist visiting Miami from the United Kingdom. Eleven (79%) case-patients resided in or were visiting Miami-Dade County, 2 (14%) resided in Broward County, and 1 (7%) was a resident of Palm Beach County. Of the 14 case-patients, the median age was 45 years (range 1–84). Eight (57%) case-patients were female, 7 (50%) were Hispanic; 8 were white, and 6 were black. Twelve patients had a bacteremia syndrome, 2 had meningitis, and 1 had pneumonia in addition to bacteremia. Four of 14 patients died, all with bacteremia. The epidemiologic investigation has not identified any common exposures, social settings, or other connections among patients in this series. Six (43%) case-patients had onset of illness within 10 days of their birthdays (binomial probability, p<0.0001), but no obvious detailed exposures were identified related to this observation.

**Table 1:** Demographic and clinical characteristics of patients with serogroup W135 meningococcal disease, southeastern Florida, USA, 2008–2009*

Case ID	Age, y/ sex	Race	Hispanic ethnicity	County	Month of onset	Onset near birthday†	Syndrome	Specimen source	Outcome	PFGE group
1	39/M	White	Y	Broward	2008 May	Y	B	Blood	Survived	1
2	1/M	White	Y	Miami-Dade	2008 Dec	Y	M	CSF	Survived	1
3	29/M	White	Y	Palm Beach	2009 Jan	N	M	CSF	Survived	1
4	83/M	White	Y	Miami-Dade	2009 Jan	Y	B, P	Blood	Died	1
5	84/F	White	Y	Miami-Dade	2009 Jan	N	B	Blood	Survived	1
6	26/F	Black	N	Miami-Dade	2009 Jan	Y	B	Blood	Survived	1
7	70/M	White	Y	Miami-Dade	2009 Feb	N	B	Blood	Survived	1
8	50/F	Black	N	Miami-Dade	2009 Feb	N	B	Blood	Survived	1
9	77/F	Black	N	Miami-Dade	2009 Feb	N	B	Blood	Survived	2
10	58/F	Black	N	Broward	2009 Mar	Y	B	Blood	Died	4
11	20/F	White	N	Miami-Dade	2009 Mar	N	B	Blood	Died	1
12	69/F	White	Y	Miami-Dade	2009 Apr	N	B	Blood	Survived	4
13	26/F	Black	N	Miami-Dade‡	2009 Apr	Y	B	Blood	Died	3
14	19/M	Black	N	Miami-Dade	2009 Apr	N	B	Blood	Survived	1

*ID, identification; PFGE, pulsed-field gel electrophoresis; B, bacteremia; M, meningitis; CSF, cerebrospinal fluid; P, pneumonia. †Within 10 days, before or after. ‡Foreign tourist visitor to Miami-Dade County.

**Figure 2:**
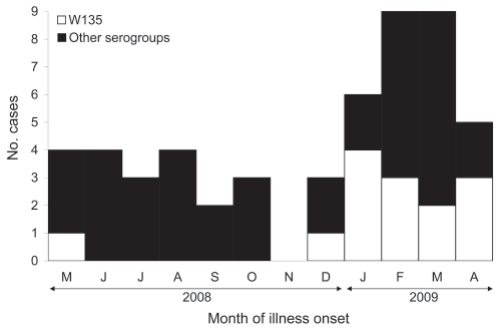
Confirmed meningococcal disease cases, by month of onset, Florida, USA, May 2008–April 2009.

Illness onset for the 14 W135 patients in this cluster is shown in relation to onset for all 38 meningococcal disease patients with other serogroups identified in Florida during the same 12-month period (Figure 2). Demographic and clinical factors were also compared between these serogroup W135 and non-W135 patients (Table 2). As a group, serogroup W135 patients were older, were more likely to be Hispanic and to reside in or be visiting the southeastern 3-county region, were more likely to have bacteremia, and had higher mortality rates than non-W135 serogroup patients. However, other than Hispanic ethnicity and residence in the southeastern region, none of these differences were significant.

**Table 2:** Characteristics of patients with serogroup W135 and non-W135 meningococcal disease, Florida, USA, May 2008–April 2009

Variable	No. positive/no. tested (%)		p value*
	W135, n = 14	Non-W135, n = 38	
Female sex	8/14 (57)	22/38 (58)	1.00
White race	8/14 (57)	30/38 (79)	0.16
Hispanic ethnicity	7/14 (50)	7/38 (18)	0.04
Residence in southeast region	14/14 (100)	12/38 (32)	<0.0001
Bacteremia	12/14 (86)	24/38 (63)	0.18
Meningitis	2/14 (14)	13/38 (34)	0.30
Pneumonia	1/14 (7)	5/38 (13)	1.00
Death	4/14 (29)	4/38 (11)	0.19

*By 2-sided Fisher exact test used for categorical variables; Wilcoxon rank-sum test used for age. Median age for W135 patients, 45 years; median age for non-W135 patients, 27 years; p value = 0.07.

Twelve isolates from the Florida cluster were forwarded to Centers for Disease Control and Prevention laboratories for antimicrobial susceptibility testing. All 12 isolates were sensitive to all antimicrobial agents tested (penicillin G, ceftriaxone, ciprofloxacin, rifampin, azithromycin). Seven of these isolates underwent additional molecular characterization with PCR and multilocus sequence typing by using methods previously described (*1**,**3*). The isolates were confirmed as serogroup W135 by real-time PCR with primers specific for serogroups A, B, C, W135, X, and Y. These isolates were also found to belong to the sequence type (ST)-11/electrophoretic type 37 clonal complex.

The dominant PFGE pattern seen in the Florida cluster is designated H46N06.0068, and is closely related to the pattern observed in the large multicountry outbreak associated with Hajj pilgrims occurring in 2000 (*1*). The 2000 outbreak involved >400 cases and was caused by a single clone of the ST-11 clonal complex. In recent years, increases in serogroup W135 have been noted in northern Argentina (*4*) and southern Brazil (*5*). Among serogroup W135 isolates in these countries, the most common clone has been the hypervirulent ST-11 complex (*4**,**5*).

From 1997 through 2006, the annual incidence of meningococcal disease in Florida declined gradually from ≈1.2 to 0.4 cases per 100,000 population (*6*). Historical trends of surveillance data in Florida suggest seasonal peaks of meningococcal disease during November–May, corresponding to the winter dry season, when tourist visitors and part-time residents are most likely to visit. The 3-county region of southeastern Florida in which this W135 cluster occurred had an estimated population in 2008 of >5.5 million inhabitants; 2.4 million of these reside in Miami-Dade County, where >60% of residents are Hispanic. During December 2008 through April 2009, the 32 total patients with meningococcal disease reported in the state represent an annual incidence of 0.41 cases/100,000 Florida residents. The 19 total cases (13 serogroup W135) in the 3-county region during this period represent an annual population incidence of 0.82 cases/100,000 residents in these counties. For the 13 total cases occurring in Miami-Dade County during this period (11 serogroup W135), the annual incidence would be 1.26 cases/100,000 residents in the county. During May 2009, no cases of serogroup W135 meningococcal disease were reported anywhere in the state. In the absence of a more narrowly defined risk group, the cluster of serogroup W135 case-patients described in this report is still far below the threshold for recommending vaccination control efforts (10 cases/100,000 over 3 months) (*7*).

## Conclusions

Southeastern Florida is considered the gateway to the Americas, with extensive social, cultural, and commercial ties to Central and South America and the Caribbean. The dominant clonal complex observed in the Florida cluster matches the dominant type recently observed in Argentina and Brazil, raising the possibility of introduction to southeastern Florida from South America. Taken together, these observations suggest the possible establishment of a clonal complex of serogroup W135 meningococci in southeastern Florida with subsequent endemic transmission.
